# Editorial: Molecular Pathogenesis of Pneumococcus

**DOI:** 10.3389/fcimb.2017.00310

**Published:** 2017-07-11

**Authors:** Guangchun Bai, Jorge E. Vidal

**Affiliations:** ^1^Department of Immunology and Microbial Disease, Albany Medical College Albany, NY, United States; ^2^Hubert Department of Global Health, Rollins School of Public Health, Emory University Atlanta, GA, United States; ^3^Emory Antibiotic Resistance Center, School of Medicine, Emory University Atlanta, GA, United States

**Keywords:** *Streptococcus pneumoniae*, pathogenesis, gene regulation, biofilm, co-infection, drug resistance, microbial, macrophages

*Streptococcus pneumoniae* has been for decades the number one bacterial killer of children worldwide, but it is also a commensal of the human nasopharynx during childhood. Although vaccination with pneumococcal conjugate vaccines has helped decrease the burden of pneumococcal disease (PD), mortality caused by this pathogen remains high. The introduction of pneumococcal vaccines has also created a niche for vaccine-escape serotypes. Moreover, the rise of multidrug-resistant clones around the world has posed a serious threat in recent years. A better understanding of pneumococcal biology and pathogenesis is needed to further reduce the burden of PD. In the current research topic, nine papers were published for this perspective.

Complex molecules of human epithelia, including the human upper and lower airways, contain N-acetylglucosamine (NAG) that can be metabolized by pneumococcus to survive and persist during colonization, and succeed during pathogenesis. Afzal et al. investigated the transcriptome of the pneumococcal strain D39 growing in media with NAG as a sole source of carbohydrates, and demonstrated upregulated transcription of operons involved in NAG uptake. They confirmed that *nagA* and *nagB* are essential for the growth of pneumococcus in the presence of NAG and found that NagR regulates genes involved in the metabolism of NAG (Afzal et al.).

Phosphorus and metal ions are essential nutrients for all living organisms (Porcheron et al., 2013). Most bacteria encode a single Pst transporter for the uptake of inorganic phosphate (Pi). However, Zheng et al. demonstrated that *S. pneumoniae* possesses two distinct Pst transporters, Pst1 and Pst2. Interestingly, Pi uptake by these two Pst proteins can be compensated by a Na^+^/Pi co-transporter, NptA, in non-encapsulated *S. pneumoniae* (NESp) strains. In contrast, either Pst1 or Pst2 is needed for the growth of encapsulated strains. The Pi uptake transporters in *S. pneumoniae* are tightly controlled by two transcription factors, PhoU1 and PhoU2, to maintain high Pi transport in different Pi availability, which is required for optimal capsule biosynthesis (Zheng et al.).

It is known that AdcR-regulated genes play a role in pneumococcal pathogenesis. Manzoor et al. found that a number of genes belonging to the AdcR and the PsaR regulons are highly upregulated in the presence of Ni^2+^, suggesting the importance of Ni^2+^ in pneumococcal virulence. Additionally, most bacteria have developed strategies for iron acquisition from the host hemoglobulin (Hb) or heme (Andrews et al., 2003). *S. pneumoniae* Spbhp-37 is a cell surface protein and has an high affinity with Hb. The expression of Spbhp-37 is upregulated when *S. pneumoniae* is grown in media where Hb is provided as the sole iron source (Romero-Espejel et al.). Therefore, Spbhp-37 might be essential for *S. pneumoniae* to establish an infection in the host.

Another surface protein of *S. pneumoniae*, pneumococcal surface protein K (PspK), is known to increase colonization and virulence by NESp during otitis media (OM) episodes (Keller et al., 2014). Therefore, PspK is considered as a NESp-specific virulence factor. Interestingly, it was demonstrated that expression of *pspK* in an encapsulated pneumococcal strain significantly increased adhesion and invasion of human epithelial cells. Additionally, Murine colonization was increased when a capsule mutant expressed *pspK* (Keller et al.). These findings indicate that PspK plays distinct roles in pathogenesis between encapsulated *S. pneumoniae* and NESp.

It is known that macrophages play an essential role in the recognition and clearance of bacterial infections by either innate immunity or present antigen to establish acquired immunity. Phosphoinositide 3-kinase (PI3K) is an essential regulator for Fcγ and CR-mediated phagocytosis (Cox et al., 1999). Protein kinase B (Akt or PKB) is one of the major signal transducers and is a downstream target of PI3K (Alessi et al., 1997). By using immuno-fluorescence microscopy, antibiotic protection assays, and addition of inhibitors of central host cell kinase, new evidence showed that PI3K and Akt play an important role in uptake and killing of *S. pneumoniae* by human THP-1 macrophages, which is independent of Fcγ or CR-mediated phagocytosis pathways (Kohler et al.).

Through investigating interactions between pneumococcal biofilms and biofilms produced by other human pathogens, Khan et al. discovered that pneumococcal strains produce a factor(s) that efficiently eradicates, i.e., within hours, *Staphylococcus aureus* biofilms including those produced by methicillin-resistant *S. aureus* (MRSA) strains. Eradication was more efficient when *S. pneumoniae* and *S. aureus* had direct contact, which mimicked the condition that the strains would naturally encounter on the human upper airways. Physical-mediated killing did not require production of hydrogen peroxide since a *S. pneumoniae* mutant in *spxB* gene, encoding for an enzyme involved in production of H_2_O_2_, was able to eradicate *S. aureus* biofilms. Authors hypothesized that research on factors allowing eradication of *S. aureus* will provide alternative therapeutics for *S. aureus* infections (Khan et al.).

Influenza pandemics and seasonal outbreaks have enhanced susceptibility to secondary infection with *S. pneumoniae*. Boianelli et al. developed a computational approach to evaluate the efficacy of treatment with Oseltamivir for flu infection and secondary pneumococcal pneumonia. Their mathematical algorithms revealed that increasing the recommended dose (i.e., 75 mg) to 150 mg, or 300 mg, almost doubles the effect of Oseltamivir against secondary bacterial infection. Other aspects of the treatment were also evaluated using their computational approach. Overall, their study warrants clinical trials to determine the efficacy of treatment regimens with Oseltamivir against lethality of secondary pneumococcal infection post influenza (Boianelli et al.).

Macrolides inhibit bacterial protein synthesis by binding to the large 50S ribosomal subunit (Schroeder and Stephens). These antibiotics have been extensively used for treatment of pneumococcal infection, which raises a strong selective pressure contributing to the expansion of macrolide-resistant *S. pneumoniae*. Macrolide resistance in *S. pneumoniae* is predominantly due to ribosomal methylation by the gene product encoded by *erm*(*B*) and macrolide efflux by a two-component efflux pump encoded by *mef* (*E*)/*mel* (Schroeder and Stephens). Current pneumococcal vaccines are effective in reducing macrolide resistance caused by vaccine serotypes. On the other hand, “serotype replacement” post vaccination might lead to macrolide resistance in new serotypes, which remains a public health concern.

## Author contributions

All authors listed have made a substantial, direct and intellectual contribution to the work, and approved it for publication.

### Conflict of interest statement

The authors declare that the research was conducted in the absence of any commercial or financial relationships that could be construed as a potential conflict of interest.
